# Assessing the risk of dengue severity using demographic information and laboratory test results with machine learning

**DOI:** 10.1371/journal.pntd.0008960

**Published:** 2020-12-23

**Authors:** Sheng-Wen Huang, Huey-Pin Tsai, Su-Jhen Hung, Wen-Chien Ko, Jen-Ren Wang

**Affiliations:** 1 National Mosquito-Borne Diseases Control Research Center, National Health Research Institutes, Tainan, Taiwan; 2 Department of Pathology, National Cheng Kung University Hospital, and College of Medicine, National Cheng Kung University, Tainan, Taiwan; 3 Department of Medical Laboratory Science and Biotechnology, National Cheng Kung University, Tainan, Taiwan; 4 Department of Internal Medicine, National Cheng Kung University Hospital, and College of Medicine, National Cheng Kung University, Tainan, Taiwan; 5 National Institute of Infectious Diseases and Vaccinology, National Health Research Institutes, Tainan, Taiwan; 6 Center of Infectious Disease and Signaling Research, National Cheng Kung University, Tainan, Taiwan; NIAID Integrated Research Facility, UNITED STATES

## Abstract

**Background:**

Dengue virus causes a wide spectrum of disease, which ranges from subclinical disease to severe dengue shock syndrome. However, estimating the risk of severe outcomes using clinical presentation or laboratory test results for rapid patient triage remains a challenge. Here, we aimed to develop prognostic models for severe dengue using machine learning, according to demographic information and clinical laboratory data of patients with dengue.

**Methodology/Principal findings:**

Out of 1,581 patients in the National Cheng Kung University Hospital with suspected dengue infections and subjected to NS1 antigen, IgM and IgG, and qRT-PCR tests, 798 patients including 138 severe cases were enrolled in the study. The primary target outcome was severe dengue. Machine learning models were trained and tested using the patient dataset that included demographic information and qualitative laboratory test results collected on day 1 when they sought medical advice. To develop prognostic models, we applied various machine learning methods, including logistic regression, random forest, gradient boosting machine, support vector classifier, and artificial neural network, and compared the performance of the methods. The artificial neural network showed the highest average discrimination area under the receiver operating characteristic curve (0.8324 ± 0.0268) and balance accuracy (0.7523 ± 0.0273). According to the model explainer that analyzed the contributions/co-contributions of the different factors, patient age and dengue NS1 antigenemia were the two most important risk factors associated with severe dengue. Additionally, co-existence of anti-dengue IgM and IgG in patients with dengue increased the probability of severe dengue.

**Conclusions/Significance:**

We developed prognostic models for the prediction of dengue severity in patients, using machine learning. The discriminative ability of the artificial neural network exhibited good performance for severe dengue prognosis. This model could help clinicians obtain a rapid prognosis during dengue outbreaks. However, the model requires further validation using external cohorts in future studies.

## Author summary

Dengue virus infects millions of people annually and is associated with a high mortality rate. When outbreaks occur, hospitals are often overcrowded with patients. Thus, novel approaches are required to accelerate patient triage for hospitalization, or further intensive care. Machine learning is being widely applied for resolving various problems, including medical diagnosis and outcome prediction. Here, we combined information from patients, including age, sex, and rapid virus test results, to develop a machine learning model for severe outcome prediction. The developed machine learning model displayed good performance for severe dengue disease prediction, and all information required for the model could be easily obtained. We also found that patients who were over 60 years old, who had detectable nonstructural protein-1 from dengue virus, or who had both detectable anti-dengue IgM and IgG antibodies in their sera, had a greater risk of progression to severe dengue. This study established a new approach to predict dengue disease outcomes by applying machine learning and defined the risk factors for severity prediction.

## Introduction

Dengue virus (DENV) causes more than 90 million acute infection cases and 0.5 million fatalities worldwide each year [1]. Dengue disease is an acute febrile disease caused by the DENV, which is transmitted from mosquitos to humans [2]. Most patients present with acute dengue fever, and approximately 5–20% of patients progress to severe dengue with bleeding, plasma leakage, shock, organ failure, and even death [3]. Four serotypes of DENV, including DENV-1 to DENV-4, have recently circulated in tropical and subtropical regions around the world [4]. Although robust antibody responses have been detected in individuals who have recovered from primary DENV infections, these antibodies only have the capacity to prevent re-infection by the same serotype (homologous serotype). Thus, individuals remain susceptible to a second infection with a different serotype (heterologous serotype), and re-infection by heterologous serotypes is known to increase the risk of severe dengue disease through antibody-dependent enhancement (ADE) of DENV [5]. Thus, the antibody response to DENV infection is both beneficial and harmful to the host.

Virological and serological methods, including testing for viral RNA, DENV nonstructural protein 1 (NS1) antigenemia, anti-dengue IgM, and antigen-dengue IgG, have been widely applied in the diagnosis of DENV infection. DENV viremia occurs for 3–5 days prior to fever onset and continues for approximately 5 days into febrile illness [6]. During viremia, viral RNA and NS1 antigen can be detected in serum or plasma samples from infected patients. Among these markers, NS1 antigen is a widely applied marker in rapid diagnosis owing to its abundance along with viral RNA on the day of disease onset in patient serum. In addition to viral RNA and NS1 antigen, the presence of anti-dengue IgM and IgG antibodies is also commonly evaluated following dengue viremia [6]. In primary DENV-infected patients, anti-dengue IgM antibodies gradually increase after the day of disease onset, and anti-dengue IgG antibodies increase after IgM antibody increases. During secondary DENV infection, anti-dengue IgM and IgG antibodies simultaneously increase after viremia [7]. Since viral RNA and NS1 antigenemia may not be detectable in patient’s serum after the acute febrile phase, either the presence of anti-dengue IgM or the elevated amount of anti-dengue IgG antibodies can be used as markers to diagnose dengue infection.

After identifying individuals who are infected by DENV, evaluating their risk of developing severe dengue becomes the key factor for disease control. Clinical presentation combined with the results of laboratory blood tests have generally been used as markers of dengue severity prognosis [8]. According to clinical presentation, the World Health Organization (WHO) announced improved criteria in 2009 [8]; based on these criteria, patients can be divided into three categories, i.e., dengue fever without warning signs, dengue with warning signs, and severe dengue. The warning signs of mild dengue disease include high fever, severe headache, pain behind the eyes, muscle and joint pain, nausea, vomiting, swollen glands, and skin rash. Patients with severe dengue further present with severe abdominal pain, persistent vomiting, rapid breathing, bleeding gums, fatigue, restlessness, and hematemesis. In addition to clinical manifestations, other markers, such as high fever, platelet depletion, comorbidities, secondary infections, hemoconcentrations, rhabdomyolysis, prolonged prothrombin time, virus serotypes, and increased viral antigen NS1 levels, have been reported to be associated with increased severity in dengue patients [9–13]. These warning signs and identified risk factors have been applied for the evaluation of the severity of dengue; however, the time required to perform additional laboratory blood tests with advanced medical devices makes it difficult to rapidly triage patients with a high risk of severe dengue for further medical treatment or hospitalization [14]. Hence, a more efficient prognostic tool is urgently required, particularly for application during major dengue outbreaks.

Artificial intelligence has recently attracted much attention in various fields of health and medicine. Different artificial intelligence and machine learning (ML) methods have been applied for various purposes, including image recognition, patient phenotyping, and outcome prediction for diseases such as cancer [15–19], cardiac arrest [20], Alzheimer’s disease [21–23], respiratory diseases [24,25], rheumatic diseases [26], cornea and retinal diseases [27,28], gastrointestinal diseases [29,30], and infectious diseases [31–35]. These studies revealed that artificial intelligence has the capacity to assist clinicians in the disease diagnosis with high efficiency and accuracy. The advantages of artificial intelligence include improved medical treatment of patients and reduced duration of diagnosis after patients are examined using medical imaging or laboratory tests. However, very few artificial intelligence-based approaches or ML methods have been developed to predict dengue severity thus far [36]. Accordingly, in this study, we retrospectively established a rapid prognosis system for severe dengue using an ML approach according to rapid diagnostic test results and demographic characteristics of the patients. Since rapid diagnostic tests are performed when patients are suspected to be infected with DENV, these test results along with patient demographic information are available without the requirement of additional tests.

## Methods

### Ethics statement

All demographic and clinical data were anonymized and de-identified prior to analysis; thus, informed consent was waived. The waiver was approved by the Institutional Review Board of National Cheng Kung University Hospital (approval no. B-ER-107-224).

### Demographic and clinical characteristics of patients with dengue

Demographic information and laboratory test results for patients with dengue were obtained from our previous study [37]. Suspected DENV-infected patients were enrolled at National Cheng Kung University Hospital between July and November 2015. Among these patients, 798 for whom all demographic information and laboratory test results were available, including disease severity, age, sex, viral RNA copies, NS1 antigen, anti-dengue IgM, and anti-dengue IgG, were enrolled in this study. Additional molecular tests for DENV serotyping were performed, and all patients in this study were confirmed to be infected with DENV-2. Patients were categorized as having mild or severe dengue by clinicians before hospital discharge (inpatients) or by the Taiwan CDC dengue surveillance system in the severe case report (outpatients), according to the 2009 WHO diagnostic criteria for severity. In brief, patients with mild dengue typically develop high-grade fever that is often accompanied by facial flushing, skin erythema, generalized body ache, myalgia, arthralgia, headache, anorexia, nausea, and vomiting, and some of these patients may have sore throat, injected pharynx, and conjunctival injection. The liver is often enlarged and tender after a few days of fever. The following criteria were used to categorize cases as severe dengue: severe plasma leakage, severe bleeding, or severe organ involvement.

### Clinical specimens

Serum samples from patients with suspected DENV infection were collected at the Clinical Virology Laboratory of National Cheng Kung University Hospital (NCKUH) between July and November 2015. Samples were screened using one-step immunochromatographic Dengue DuoDengue NS1 Ag + Ab Combo assays (SD BIOLINE, Yongin, Republic of Korea) according to the manufacturer’s instructions for qualitative antigen and antibody detection. For estimating DENV viral loads, viral RNA copies were amplified using LightMix dengue virus EC quantitative reverse transcription polymerase chain reaction, as previously described [37]. In brief, viral RNA was extracted from serum samples and an extraction control sample using QAIamp viral RNA mini kit (Qiagen, Venlo, Netherlands) or the automated extraction system, LabTurbo Virus mini Kit within the LabTurbo 48 Compact System (Taigen BioscienceCorp., Taipei, Taiwan). qRT-PCR analysis of viral loads was performed using LightMix dengue virus EC kit (qRT-PCR; TIB Molbiol, Berlin, Germany), which is capable of identifying all four dengue serotypes. cDNA was generated from the RNA samples using a FirstStrand cDNA Synthesis kit (Roche, Basel, Switzerland) and a DENV-specific primer included in the LightMix dengue virus EC kit. The qRT-PCR assay was then performed in a LightCycler 2.0 or LightCycler 480 II device (Roche, Basel, Switzerland), according to the manufacturer’s instructions. The LightMix dengue virus EC kit provides cloned dengue DNA at concentrations of 10^1^ to 10^6^ copies/reaction as standards. The cycle number of the Crossing Point (Cp) of each sample was calculated automatically by the Second Derivative Maximum method (Automated (F" max)) using the LightCycler software. A logarithmic transformation was applied to all resulting data. The amount of virus per sample (viral load) was reported in copies/reaction, as automatically generated by the LightCycler software.

### Statistical and descriptive analysis

To identify the characteristics that contribute to disease severity, we analyzed the contribution of demographic information and laboratory test results of DENV-2 infected patients. Both age and viral RNA amount were compared using Student’s *t*-test. Categorical characteristics, including sex, DENV NS1, anti-DENV IgG, and anti-DENV IgM test results, were compared with DENV disease severity using the Chi-squared test of independence. We further performed multivariate logistic regression (LR) to evaluate the association of demographic information (age and sex) and clinical test results (NS1, IgM, IgG) with the prognosis of severe dengue using R version 3.4.4. To analyze the age variable more intuitively, we further categorized age into two groups. As the average age among patients was 55.8 years, we chose 50 years as a cut-off to categorize cases for multivariate logistic regression analysis. We determined the univariate and multivariate-adjusted odds ratio (OR) and 95% confidence interval (CI) for each factor to predict severity using LR. The factors that exhibited *P* < 0.05 from this analysis were considered significantly associated with severe dengue.

### ML models and preprocessing

The ML models and preprocessing strategy were adapted from a previous report by Nanayakkara et al. [20]. We used LR, support vector machine (SVM), random forest (RF), gradient boosting machine (GBM), and artificial neural network (ANN) methods, which are commonly used for binary classification questions in medicine, to establish patterns for distinguishing severe cases from mild cases [20]. Briefly, RFs first established multiple decision trees and generated various divisions of the data to retrieve an output [38]. Decision trees selected these divisions based on minimizing impurity. Similar to RFs, GBMs first trained the models in a gradual, additive, and sequential manner and collected weak decision trees [38]. Through a process of iteratively training new models to improve the weakly classified observations of the previous models, GBMs assembled these models together and made predictions. SVMs created a line or a hyperplane to divide the data into different classes to address the predictions [38]. Through imitating biological neurons of the human brain, the ANN method then established multiple hidden layers of neurons that link inputs to the output neuron [39]. Models were then constructed using open-source software and its libraries, including Scikit-learn 0.22.2 [40], Tensorflow 2.0.0 [41], and Python 3.6.10 [42].

To validate the prognosis performance of the models, we used a stratified 10-fold cross-validation approach with training and testing datasets. In this approach, the original sample was randomly partitioned into 10 equal size subsamples. Of the 10 subsamples, a single subsample was retained as the validation data for testing the model, and the remaining 9 subsamples were used as training data. The folds were selected so that each fold contains roughly the same proportions of severe and mild case labels. In a 90% and 10% training-testing set, each subsample contained 80 cases, including 14 severe dengue cases. We then repeated this process 10 times (the folds), with each of the 10 subsamples being used the validation data, exactly once. In each instance of partition, the remaining training data was used to develop the prognosis model. To fine-tune the parameters of the prognosis model, we used either grid or random hyperparameter searches to search for optimal hyperparameters for each model by another stratified 10-fold cross validation process using the training data. A broad range of hyperparameters was applied in the parameter search, and the area under the receiver operating characteristic curve (AUROC) was referred to as the optimization metric. The AUROC of the model was assessed using the logarithmic loss function. The age variable was normalized by 100 to yield values between 0 and 1. Feature normalization is beneficial for improving the numerical stability of the model and often reduces training time because similar ranges of values help to quickly converge the gradient descents during model training. The performance of the established models was validated by receiver operating characteristic (ROC) curves, which summarized the trade-off between the true- and false-positive rates, and the AUROC was calculated to validate the performance of the established models. We also compared the performance of the models balance accuracy values based on the average recall obtained on severe and mild case groups. The 10 AUROC and balance accuracy values from each fold were then averaged for model performance validation.

### Model explanation methodology

To identify potentially relevant features on a per-patient basis, we assessed explainability using SHapley Additive exPlanations (SHAP). This method has been previously described in detail [43] and connects game theory with local explanations, uniting several previous methods. Briefly, SHAP generates a locally interpretable model for individual prediction from a complex model using an explainer method that combines the inputs together to evaluate the effects on the predictive model. Assessing how the ML model makes predictions is essential, particularly for predictive models with good performance; however, the ML model often achieved good performance by using complex models, which are difficult to explain. Thus, to explain the developed ANN model, we applied the SHAP model explainer [43], which is a unified framework to interpret predictions generated by our model. In the model explanation, we used a stratified holdout approach by randomly partitioning patients into subsets with 90% and 10% of training and testing datasets, with the same proportion of severe and mild cases in training and testing datasets. The training dataset was used to establish the ANN with hyperparameter tuning via stratified 10-fold cross validation. The testing dataset was analyzed using the SHAP explainer, which illustrated the output of ML models by assigning an importance value (the SHAP value) to each feature for prediction. This also included the identification of a new class of additive feature importance measures as well as theoretical results. The SHAP explainer could interpret for all predictions, whether each feature increased (SHAP value > 0) or decreased (SHAP value < 0) the potency for different classification results. Additionally, the SHAP explainer improved computational performance and consistency with higher local accuracy than other model explainer approaches [43]. The summary plot demonstrates how the SHAP values vary along with the feature values (age) and the designated values (NS1, anti-DENV IgM, and anti-DENV IgG: positive as 1 and negative as 0; sex: male as 1 and female as 0). The dependence plot reveals the combined effects of two features on the SHAP values. In the dependence plots, features that were most strongly associated with each other were paired as a “feature pair” for which the combined effects on disease severity prognosis were examined.

## Results

### Statistics and machine learning models using demographic information and laboratory test results

To develop a rapid prognostic model to predict severe dengue, we retrospectively analyzed the profiles of DENV-infected patients from the 2015 Taiwan outbreak. Among 798 recruited patients, 17.4% (138) of cases had severe dengue, and 82.6% (660) of cases had mild dengue. Patients with DENV were evaluated for presence of DENV NS1 antigen, anti-DENV IgG, anti-DENV IgM, and viral RNA in serum samples.

To identify the characteristics associated with disease severity that should be included as features of the prognostic model, we first analyzed association of the characteristics, including age, sex, DENV NS1 antigen, viral RNA amount, and anti-DENV IgG and IgM antibodies, with the severity of dengue (Table 1). The results indicated that patients with severe dengue were significantly older than patients with mild dengue (median age: 75 versus 55 years, respectively; *p* < 0.001). Additionally, more severe cases exhibited detectable DENV NS1 antigen (94.9% versus 74.2%, respectively; *p* < 0.001), anti-DENV IgG antibody (30.2% versus 23.6%, respectively; *p* < 0.001), and anti-DENV IgM antibody (25.8% versus 24.3%, respectively; *p* = 0.008) compared with mild cases in serum samples collected on day 1 when they sought medical advice in our hospital during acute febrile illness. In contrast, neither patient sex nor viral RNA amount in serum was significantly associated with disease severity. In summary, patient characteristics, including age, serum DENV NS1 antigen, and anti-DENV IgG antibody levels, were associated with disease severity in DENV-2-infected patients. This implies that characteristics may be good predictors of disease severity

**Table 1:** Demographic information and laboratory test results of DENV-2-infected patients. Age is reported as the median with interquartile range shown in square brackets. The viral RNA amount is reported as the mean with standard deviation in parentheses. For the remaining variables, the percentages of total number of patients are given in parentheses.

Characteristics	Severe dengue (n = 138)	Mild dengue (n = 660)	*P* value
Age (years)	75 [68–79]	55 [35–71]	< 0.001
Male sex	76 (54.6%)	342 (51.8%)	0.486
DENV NS1 positive	132 (94.9%)	490 (74.2%)	< 0.001
Viral RNA amount (copies/140 μL serum)	1.72 × 10^7^ (4.93 × 10^7^)	1.03 × 10^7^ (3.97 × 10^7^)	0.123
Anti-DENV IgG positive	42 (30.2%)	156 (23.6%)	< 0.001
Anti-DENV IgM positive	36 (25.8%)	161 (24.3%)	0.008

To develop a prognosis model for rapid triage, we next excluded viral RNA amount, which result have to be retrieved with need for advanced medical instrument, and applied the patient demographic characteristics, and their laboratory test results, including age, sex, DENV NS1 antigen, anti-DENV IgG antibody, and anti-DENV IgM antibody for model development. First, we used the multivariate LR method to assess the impact of clinical and virological/serological laboratory characteristics on DENV disease severity (Table 2). Among the evaluated characteristics, age over 50 years (univariate OR: 7.22, 95% CI: 4.14–13.66; multivariate-adjusted OR: 7.58, 95% CI: 4.28–14.54; *p* < 0.001) and presence of NS1 (NS1 +) in the serum (univariate OR: 7.63, 95% CI: 3.60–19.74; multivariate-adjusted OR: 10.07, 95% CI: 4.61–26.58; *p* < 0.001), were the two major risk factors for severe DENV disease.

**Table 2:** Multivariate logistic regression model of clinical and laboratory diagnosis characteristics for dengue virus type 2 infection.

Characteristic	Severe (n = 138)	Mild (n = 660)	Univariate OR (95% CI)	*P* value	Multivariate-adjusted OR (95% CI)	*P* value
Age ≥ 50 years	377	125	7.22 (4.14–13.66)	< 0.001	7.58 (4.28–14.54)	< 0.001
DENV NS1 positive	176	622	7.63 (3.60–19.74)	< 0.001	10.07 (4.61–26.58)	< 0.001
Anti-DENV IgM positive	36	161	1.09 (0.71–1.65)	0.68	1.37 (0.82–2.28)	0.22
Anti-DENV IgG positive	42	156	1.41 (0.94–2.11)	0.09	1.15 (0.70–1.87)	0.57
Male	76	342	1.14 (0.79–1.65)	0.49	1.11 (0.75–1.65)	0.61

In addition to the multivariate LR method, we established ML models to predict the probability of patients progressing to severe dengue. The ML approach used diverse computational methods capable of incrementally building an accurate data model according to a measure of how well the model supported a given task, which may be applied for diagnosis classification in medicine. We thus utilized various ML methods, including LR, SVM, RF, GBM, and ANN, to establish models for severe dengue prognosis (Table 3). In addition to model development, we searched the hyperparameters to fine-tune each ML method for severity prognosis with high prognosis performance.

**Table 3:** Comparison of machine learning and deep learning models by 10-fold cross-validation for disease severity prediction in dengue virus type 2 infection.

Values	Logistic regression	Support vector machine	Random forest	Gradient boosting machine	Artificial neural network
Area under receiver operating characteristic curve (AUROC)	0.8152 ± 0.0718	0.8233 ± 0.0624	0.8187 ± 0.0543	0.8104 ± 0.0594	0.8324 ± 0.0268
Balance accuracy	0.5425 ± 0.0468	0.7485 ± 0.0688	0.5448 ± 0.0543	0.5867 ± 0.0712	0.7523 ± 0.0273

Values are shown as mean ± standard deviation.

**Fig 1:**
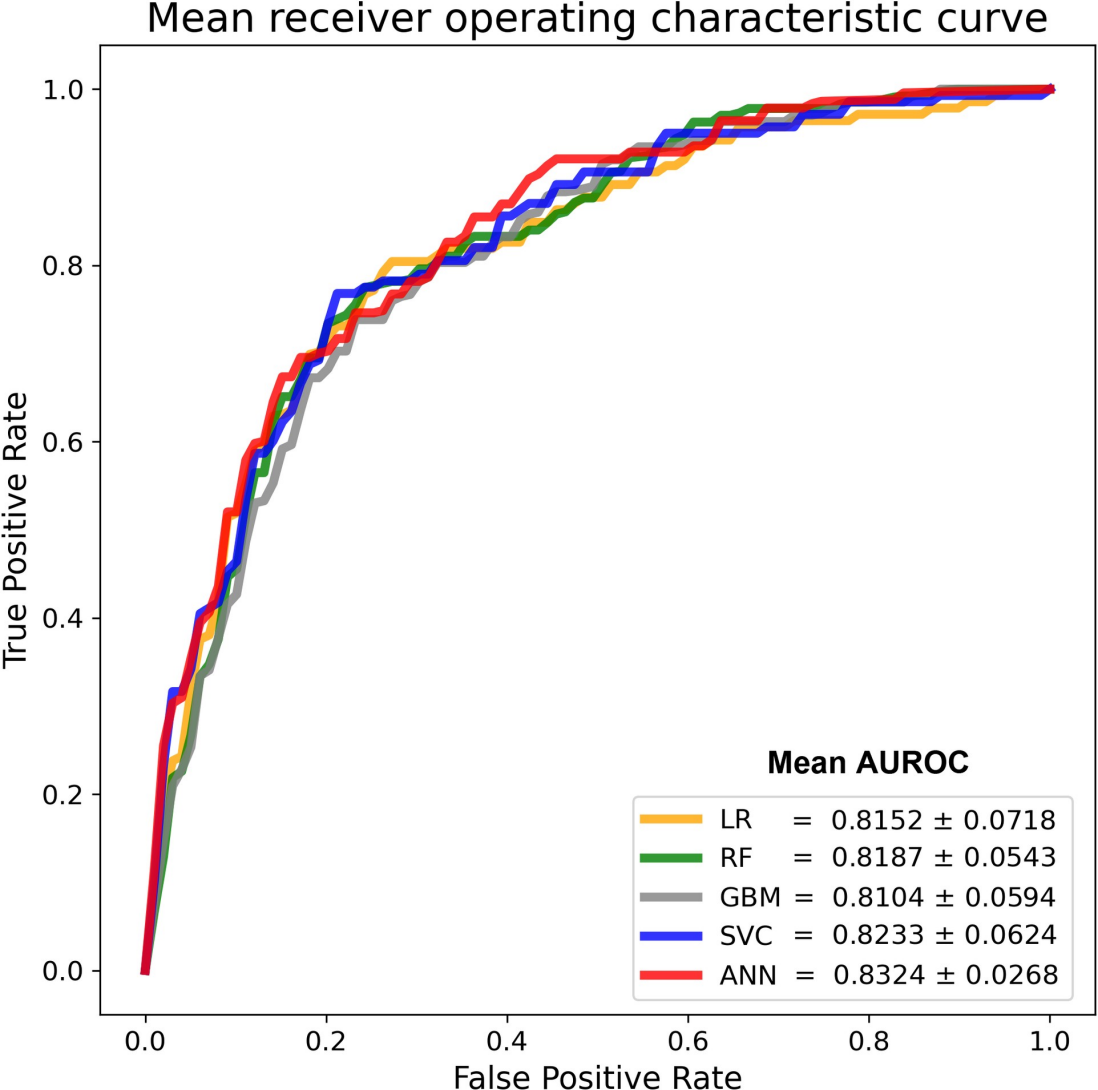
Comparing receiver operating characteristic curves of machine learning methods with 10-fold cross-validation. Mean receiver operating characteristic curves of prognostic models established using machine learning methods are displayed in the indicated colors.

To evaluate whether the ANN model was not overfitting and thus generalized to unseen data, we used a 10-fold cross-validation scheme to assess the performance of the models (Fig 1 and Table 3). The performance values of the model are not single iterations; they are the averages across ten validations of the 10-fold cross-validation process. The ANN classified the clinical outcomes of patients with slightly higher AUROC (0.8324 ± 0.0268) and balance accuracy (0.7523 ± 0.0275), compared with the LR, SVM, RF, and GBM methods (Table 3). Thus, the cross-validation results of our prognostic models for dengue disease severity revealed that utilization of the ANN method yielded a model with good prediction performance, as well as high AUROC and balance accuracy values. Ideally, a prognostic system should use readily available data for decision-making [20]. In the large 2015 Taiwan outbreak, we applied the rapid SD BIOLINE Dengue Duo kit for dengue diagnosis, and the results of NS1, IgM, and IgG were available within hours for patients in the emergency department of our hospital [37]. Since all of the characteristics used in the prognostic model, including age, sex, NS1 antigen, anti-DENV IgM, and anti-DENV IgG, could be evaluated quickly after rapid tests were performed, the ANN model that we developed can assist with and accelerate the triage of patients when hospitals are overcrowded during large outbreaks.

### Model explainability for the prognostic ANN model

**Fig 2:**
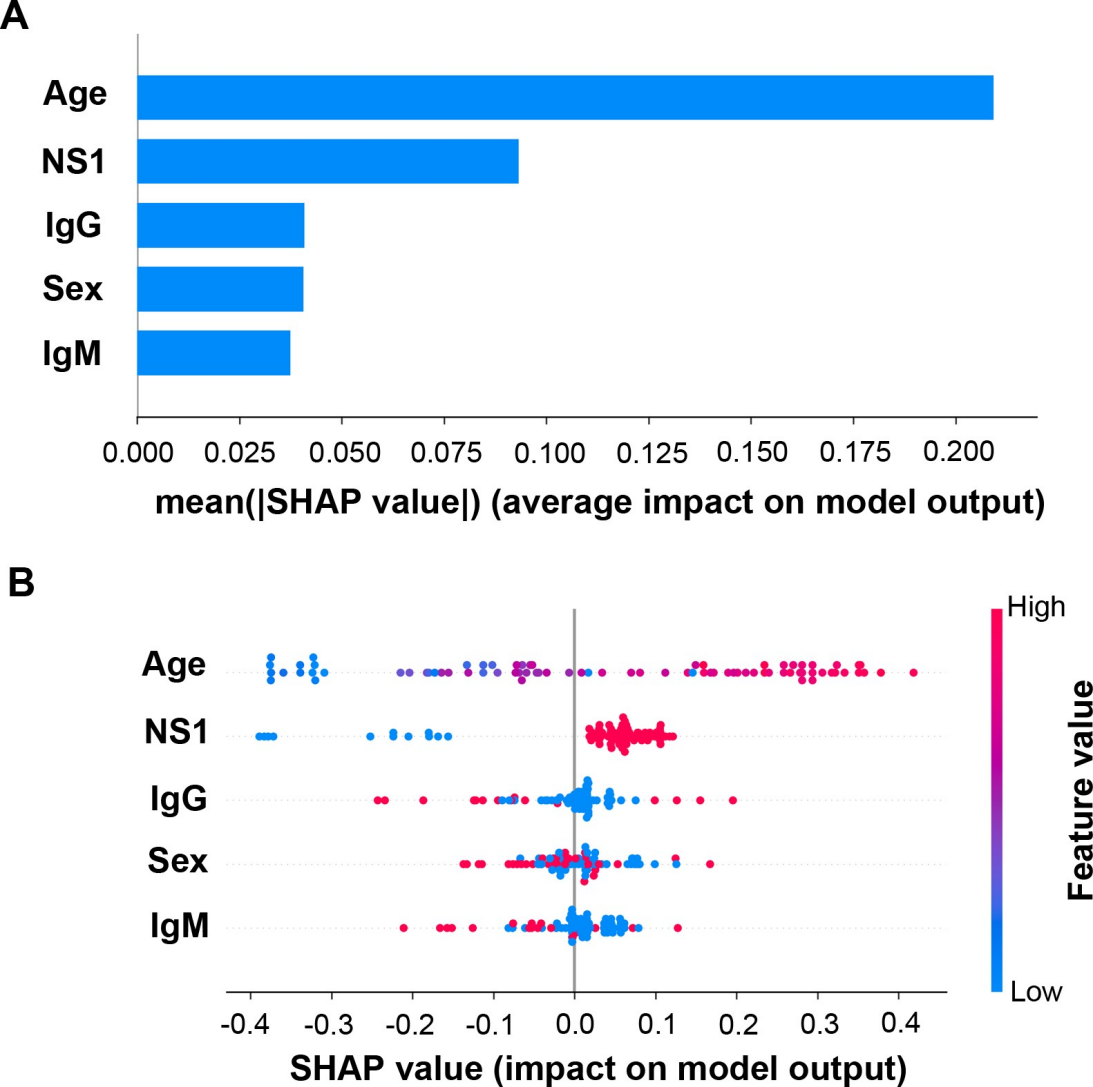
Importance plot and summary dot plot of the ANN model using SHAP explainer. (A) Importance plot depicting the features most effective at predicting dengue disease outcomes. (B) Summary dot plot mapping the effects of the indicated features on prediction outcomes, which assigns features to continuous color for age, and discrete color for other features. For NS1, anti-DENV IgM, and anti-DENV IgG, positive is red, and negative is blue; For sex, male is red, and female is blue.

Since cross-validation results indicated that the ANN method exhibited a relatively better average performance compared with the other methods, we next analyzed the ANN model to determine how the models classified disease severity by utilizing the SHAP program. Similar to the statistic and multivariate LR model results (Tables 1 and 2), the importance plot of the SHAP program showed that age and NS1 antigen were more important features than sex, anti-DENV IgM, and anti-DENV IgG (Fig 2A), although the multivariate LR model indicated that NS1 antigen had a higher OR than patient ages (Table 2). Taken together, these results confirm the importance of these specific factors in DENV disease severity.

**Fig 3:**
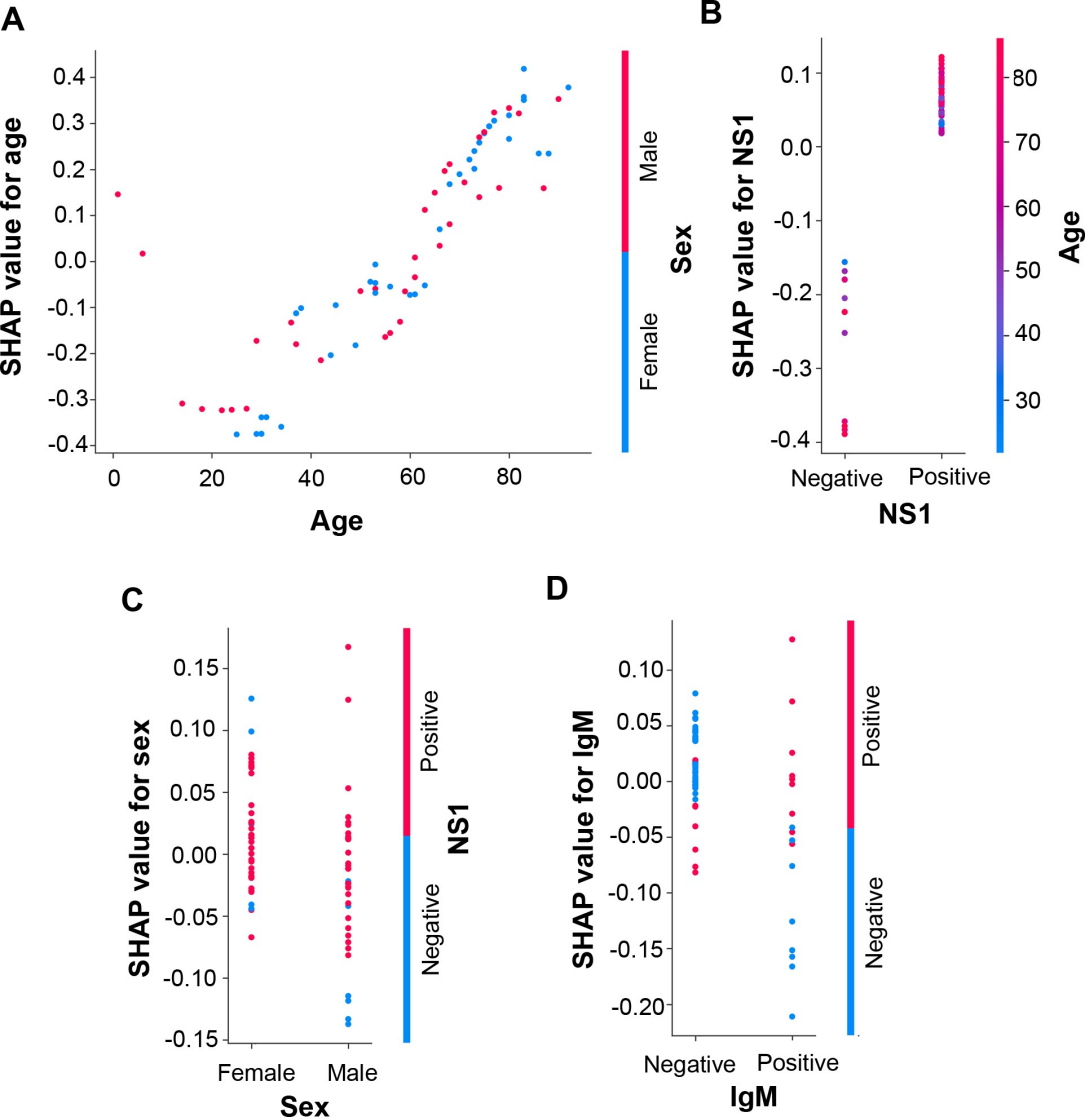
Dependence plots of the ANN model using SHAP explainer. Dependence plots of the ANN model showed the potential cooperative effects of feature pairs on prediction outcomes. For each feature, its most associated feature was chosen to be analyzed using dependence plots. The feature pairs included (A) age and sex, (B) NS1 and age, (C) sex and IgM, and (D) IgM and IgG.

Next, we analyzed the contribution of each feature to the prediction using summary (Fig 2B) and dependence plots (Fig 3) of SHAP values. In the age panel of the summary plot, the SHAP value gradually increased as patient age increased (Figs 2B and 3A). In the independence plot of age, the SHAP values changed from negative to positive as the age increased to greater than 60 years, suggesting that age greater than 60 years increased the probability of the patient progressing to severe dengue (Fig 3A). NS1 antigen was another feature that positively contributed to the SHAP value (Figs 2B and 3B). In the summary plot, we observed that the appearance of NS1 antigen increased the probability of severe dengue as an outcome prediction when evaluated using the ANN model (Fig 2B). Meanwhile, SHAP values were not directly correlated with sex, anti-DENV IgM antibodies, or anti-DENV IgG antibodies (Figs 2B, 3C and 3D).

To further assess whether dual features cooperatively affected disease severity, we next determined the effects of the feature pairs on the SHAP value to evaluate the associations between features using dependence plots (Fig 3). In the dependence plots of age, NS1, and sex, we did not observe obvious association patterns within feature pairs; thus age, NS1, and sex were considered to be independent of the other features in the ANN model prognosis (Fig 3A–3C). In contrast, we found that IgM and IgG double-positive (IgM+/IgG+) was associated with positive SHAP values in the dependence plot of anti-DENV IgM antibody (IgM) and anti-DENV IgG antibody (IgG) (Fig 3D). IgM negative and IgG positive (IgM-/IgG+), or IgM positive and IgG negative (IgM+/IgG-) generally yielded negative SHAP values. (Fig 3D).

**Fig 4:**
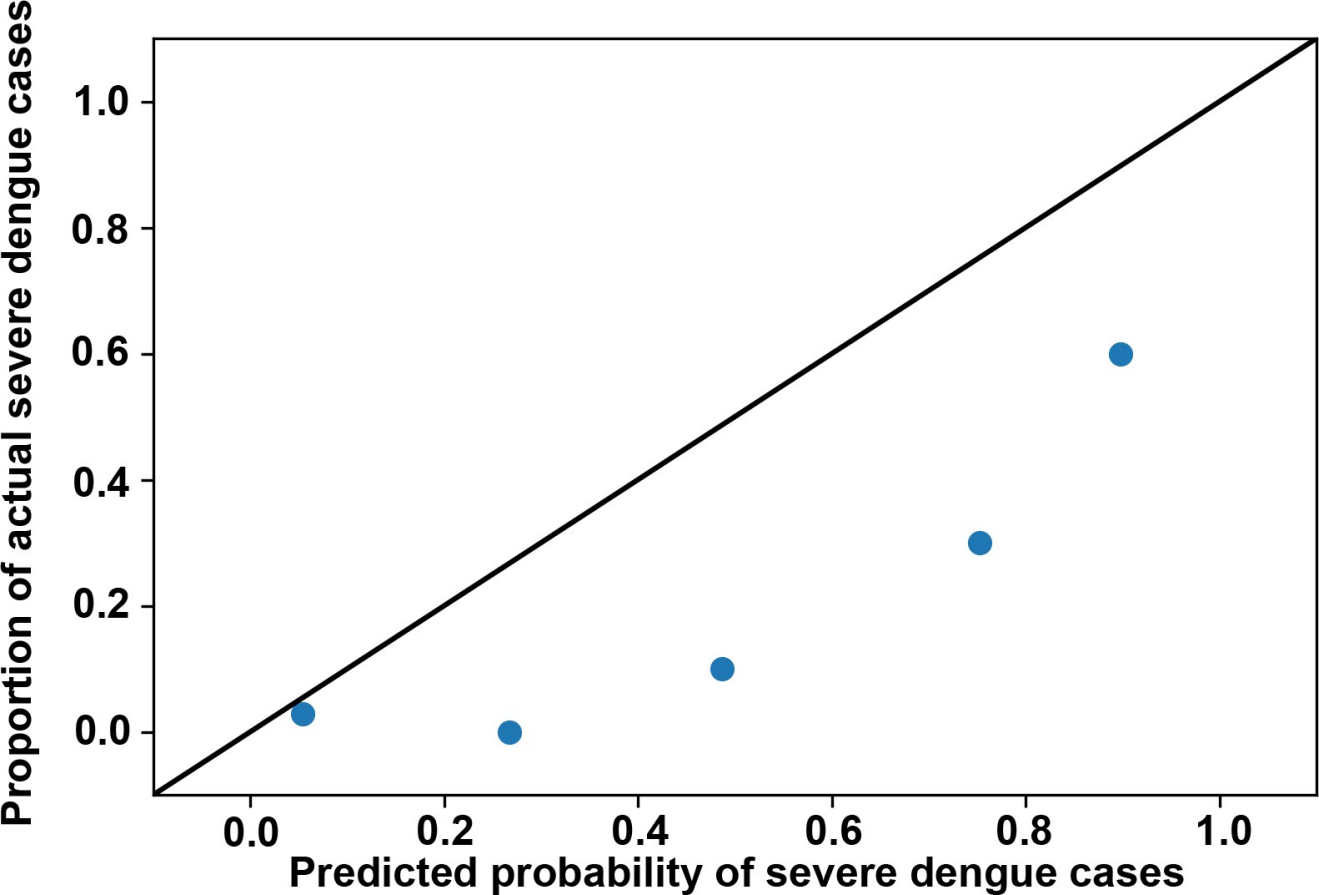
Calibration plot for the ANN model prediction. For the ANN model, the predicted probability of severe dengue cases is compared to the actual proportion of severe dengue cases among subjects in the 90–10 holdout test set. Perfect predictive ability is represented by the dashed diagonal line.

In addition to potential decision rules in the prediction, analyzing the characteristics of misclassified cases helped us to determine the limitations of the ANN model. We thus analyzed the 24 misclassified cases (Table 4), including two severe cases and 22 mild cases; these cases were incorrectly classified by the ANN model among the 80 cases of the 90:10 hold-out test dataset. The two false-negative cases were female, aged 56 and 63 years. They had NS1 antigenemia, with no anti-DENV IgM or IgG antibodies. Among the 22 false-positive cases, nine were male and 13 were female, 19 of whom were older than 60 years. Twenty-one false-positive cases were NS1 positive. In addition, two cases had NS1 antigenemia and anti-DENV IgG antibodies; three cases simultaneously had NS1 antigenemia, anti-DENV IgM, and anti-DENV IgG antibodies. The calibration plot for the prediction is displayed in Fig 4. The figure shows under-prediction of severe dengue risk among the cases of the ANN model. Misclassifications, particularly the cases that were falsely classified as negative by the ANN model, should be noted. Such cases included two females with NS1 antigenemia but no anti-DENV IgM and IgG antibodies.

**Table 4:** Definitions of false-negative and false-positive severe dengue cases in the test dataset, using ANN model prediction.

	Severity	Sex	Age	NS1 antigen	Anti-DENV IgM	Anti-DENV IgG	Prediction probability of severe cases using the ANN model
False-negative cases							
	Severe	Female	56	Positive	Negative	Negative	0.3872
	Severe	Female	63	Positive	Negative	Negative	0.2694
False-positive cases							
	Mild	Female	72	Positive	Negative	Negative	0.6373
	Mild	Female	52	Positive	Positive	Positive	0.6311
	Mild	Female	83	Positive	Negative	Positive	0.7466
	Mild	Female	53	Positive	Positive	Positive	0.6272
	Mild	Female	83	Positive	Negative	Negative	0.8125
	Mild	Male	67	Positive	Negative	Negative	0.7621
	Mild	Male	77	Positive	Negative	Positive	0.5912
	Mild	Male	65	Positive	Negative	Negative	0.7412
	Mild	Male	75	Positive	Negative	Negative	0.7996
	Mild	Female	77	Positive	Negative	Negative	0.7504
	Mild	Male	63	Positive	Positive	Positive	0.8439
	Mild	Female	76	Positive	Negative	Negative	0.7349
	Mild	Male	1	Positive	Negative	Negative	0.9256
	Mild	Female	70	Positive	Negative	Negative	0.5919
	Mild	Male	61	Positive	Negative	Negative	0.5275
	Mild	Female	83	Positive	Negative	Negative	0.8125
	Mild	Female	80	Positive	Negative	Positive	0.6627
	Mild	Female	73	Positive	Negative	Negative	0.6639
	Mild	Female	73	Negative	Negative	Negative	0.5512
	Mild	Female	75	Positive	Negative	Negative	0.7159
	Mild	Male	68	Positive	Negative	Negative	0.7679
	Mild	Male	75	Positive	Negative	Negative	0.7996

## Discussion

Since DENV activity is dramatically increased during large outbreaks, a large number of patients suspected of having dengue will seek medical advice at hospitals, particularly from the emergency department. To help accelerate triage in overcrowded emergency rooms or out-patient clinics, we developed ML models for dengue severity prognosis according to demographic information (age and sex) and laboratory test results (NS1 antigen, anti-DENV IgM antibody, and anti-DENV IgG antibody). The established ANN model predicted disease severity with good performance, and the SHAP explanation showed that patient age and the presence of NS1 antigen in serum samples were two major factors for severe dengue prediction. When we combined sex and anti-DENV IgM/IgG antibody in the serum, the ANN model could quickly predict severity using the NS1 antigen and anti-DENV IgM/IgG rapid tests. This method could enable clinicians to determine the prognosis of patients with dengue by analyzing retrieved information with a laptop, without the need for advanced medical instruments. Moreover, this approach can be applied in not only hospitals but also local clinics, which will help in identifying patients who may need further medical treatment or care.

According to the patient demographic information and laboratory test result dataset used in this study, patient age and NS1 antigenemia were the two factors that were most important for prediction. Regarding age, the SHAP values for severe dengue increased with increasing age and changed from negative to positive as age increased to greater than 60 years, suggesting that patients in this advanced age category had a higher probability of progressing to severe dengue. A potential explanation for this scenario is the gradual increase in seroprevalence rates with age in Taiwan [44]. According to a previous study, these rates in Taiwan increased significantly from 2.1% in the 30–39-year age group to 17.1% in the 60–69-year age group, and 50% in the 70–79-year age group [44]. The increasing seroprevalence rate from the younger group (30–39 years) to the older group (> 60 years) implied the low prevalence of DENV in Taiwan. Most individuals in the aged group were thought to be monotypically immune as they had been previously infected with DENV. Thus, the monotypically immune individuals in the aged group were at a high risk of secondary infection by heterologous DENV serotypes, which could induce ADE or immune mimicry in dengue infection and cause immunopathogenesis-related damage to endothelial cells, thereby causing severe plasma leakage [45–49]. This may explain the elevated potential for disease severity in the aged group. Moreover, since monotypically immune individuals were then exposed to infection by a homologous DENV serotype, they may be asymptomatic or exhibit mild dengue disease owing to the protective effects of pre-existing anti-DENV IgG antibodies [5]. However, ADE with aberrant activation of cross-reactive T cells causes severe dengue, such as dengue hemorrhagic fever and dengue shock syndrome, when monotypically immune individuals are infected with heterologous DENV serotypes [45–49]. A similar explanation can be applied to the findings of the model explainer, which indicated that IgM+/IgG+ marginally increased disease severity, but IgM-/IgG+ or IgM+/IgG- reduced disease severity. Most specimens were collected on day 1 when they sought medical advice in clinics or emergency departments of our hospital during acute febrile illness. Therefore, the simultaneous elevation of IgM and IgG (IgM+/IgG+) in the acute febrile phase of infection implied that the patients were immune to monotypic DENV and were recently infected with a heterologous serotype, which could increase the risk of progression to severe disease. Considering that the antibody, which was developed from a monotypic immune response, could protect individuals from re-infection by the homologous serotype [5], the scenario of IgM-/IgG+ in patients during the acute infection phase implied that the patients had been infected by the homologous serotype virus, which stimulated a pre-existing subset of plasma cells or memory B cells to produce anti-DENV IgG antibodies against the virus. The scenario of IgM+/IgG- in patients with acute infection may imply that they were primarily infected with DENV and that their naïve B cells have begun to produce anti-DENV IgM antibodies against DENV infection. Hence, both IgM-/IgG+ and IgM+/IgG- likely indicated that the individuals would potentially not be re-infected by the heterologous serotype, which may have caused mild dengue disease. While the information of patients regarding primary or secondary infection was not available in our de-identified data, it might be worthwhile to investigate the association between the rapid IgM/IgG test results and primary/secondary infection among dengue patients in the future.

Another important risk factor was NS1 antigenemia. NS1 antigen has been widely applied as a diagnosis marker in dengue rapid tests and identified as a virulence factor in DENV infection [50,51]. By disrupting the endothelial glycocalyx, NS1 causes vascular leakage and increases endothelial permeability [50], which can lead to plasma leakage or hemorrhage. High amounts of NS1 antigenemia have been suggested as an essential factor for severe dengue, particularly in the endemic population [52–56]. Thus, these reports implied that NS1 causes the pathogenesis of dengue infection and is associated with disease severity. Similar to previous studies, the SHAP explainer for the developed ANN model showed high SHAP values for the NS1 antigenemia group, suggesting greater risk of severity among NS1 positive patients.

In addition to the characteristics analyzed in this study, several studies have reported other potential markers in patient serum, such as immune activation markers (interleukin [IL]-6, IL-10, interferon-γ, macrophage inhibitory factor, and C-C chemokine motif ligand-4), endothelial activation markers (increase levels of angiotensin [Ang]-2, von Willibrand factor, circulating vascular adhesion molecule, vascular endothelial growth factor [VEGF], and VEGF receptor [VEGFR] 1 and decrease levels of Ang-1, a disintegrin and metalloproteinase with a thrombospondin type 1 motif member 13, and VEGFRII), and biochemical markers (increased levels of lipopolysaccharide, aspartate aminotransferase [AST], and alanine aminotransferase [ALT] and decreased levels of lipids, inhibitor alpha inhibitor protein, and nitric oxide), as predictors of severe dengue disease [57]. In addition to the soluble markers in patient serum, recent investigations have identified biomarkers associated with severe dengue development via microarray-based analysis to analyze host gene expression in peripheral blood samples from patients with different disease severities [57–67]. By combining results retrieved from patients, including demographic characteristics, dengue warning signs, other symptoms or signs, and laboratory features associated with severe dengue, researchers have further applied scoring systems to evaluate the potential for severe dengue with high performance [36,58,68]. In contrast, our prediction system applied patient information, including demographic characteristics (age and sex) and laboratory test results (NS1 antigen and anti-DENV IgM/IgG antibodies) obtained from rapid tests. Dengue NS1 and anti-DENV IgM/IgG rapid tests have been applied in the laboratory diagnosis of dengue infection, particularly during outbreaks and epidemics [37]. Since these tests are potentially conducted to diagnose dengue infection in suspected cases at clinics, hospitals, and medical centers, NS1 antigen and anti-DENV IgM/IgG antibody test results can be rapidly available as diagnostic factors for decision-making, without the need for special medical devices to detect or quantify other markers in the blood. The data can then be analyzed using a laptop to determine severity prediction using the ANN model. Through the ANN prediction system, the risk of patients progressing to severe dengue can be rapidly estimated. Although our prognostic approach exhibited marginally lower performance in terms of AUROC values compared to those reported in previous studies [58,68], the developed ANN prognostic system could rapidly generate predictions within hours, including the durations of collecting blood, performing rapid tests, and running the data prediction model. In addition, we believed that more patients’ blood test results and dengue disease histories may improve the sensitivity and decrease the false positive rate of this method. Thus, by combining demographic and laboratory test results, we can simultaneously diagnose DENV infection and predict disease outcomes using the ANN method without the requirement for additional medical laboratory instruments. This could facilitate the widespread use of this prognostic approach in hospitals and local clinics. Accordingly, by utilizing rapid dengue detection tests and the ML method, our ANN model system may improve and accelerate patient triage and therefore reduce morbidity and mortality during dengue outbreaks.

Nevertheless, several issues remain that must be explored when applying the ANN prognostic model. First, we only used a single type of rapid test kit in this study. A previous report showed that various commercial dengue rapid test kits exhibit differences in sensitivity and specificity [69]. Thus, the sensitivity, specificity, and accuracy of the ANN model will need to be further verified using different rapid test kits. Second, the age of patients with severe dengue varies among countries and regions. We recognize that our dataset included only Taiwanese patients. Most patients with severe disease in our study were older than 60 years; however, in southeastern Asia [60] and South America [56], the most severe cases are found in children. These differences in age distribution among severe cases in various endemic or outbreak regions may be the result of the prevalence of monotypic immune responses in the studied populations. Therefore, it is necessary to rebuild a new ML model when applying the prognostic system to a different population. Third, the predominant serotype or strain that appears during outbreaks of severe dengue may vary according to the region. In Taiwan, we faced two large outbreaks in 2014 and 2015. DENV-1 caused the first wave in 2014, with a mortality rate of 0.127% (15,732 cases and 20 fatalities); however, DENV-2 caused a large outbreak in 2015, with a mortality of 0.497% (43,784 cases with 218 fatalities), which was more than 3-fold higher than that of the 2014 DENV-1 outbreak. The different mortality rates between the two outbreaks, which were caused by different predominant strains in the same population, indicated that the virulence of different DENV serotypes may vary. Because our retrospective study used data retrieved from the DENV-2 outbreak in 2015 in Taiwan, our findings will need to be verified for other serotypes. Four, most severe dengue cases were observed in our hospital, the largest medical center of Tainan city, which was overcrowded during the 2015 outbreak. Although our hospital served the entire population of Taiwan, more severe cases might be recruited preferentially into this study, which might result in sampling bias. Finally, as a retrospective study, our findings still need to be validated in a prospective cohort before the prognostic model could be applied in the clinical setting.

In conclusion, we established an ML ANN model for dengue outcome prediction based on demographic information and patient laboratory test results. Upon examining the prognosis performance of the established models, we showed that the ML model developed by us using the ANN method demonstrated superior performance in severe dengue prognosis than established models. Therefore, this new prognostic model can assist physicians in evaluating the risk of patients progressing to severe dengue and can increase the efficiency of patient triage into hospitals, particularly in overcrowded medical facilities during dengue outbreaks. Since all information used in this model was available upon initial dengue infection diagnosis, the prognostic system may be widely applied by all medical institutes, particularly local clinics, for the prediction of dengue disease outcomes.
